# Employing fasting plasma glucose to safely limit the use of oral glucose tolerance tests in pregnancy: a pooled analysis of four Norwegian studies

**DOI:** 10.3389/fendo.2023.1278523

**Published:** 2023-11-30

**Authors:** Anam Shakil Rai, Line Sletner, Anne Karen Jenum, Nina Cecilie Øverby, Signe Nilssen Stafne, Elisabeth Qvigstad, Are Hugo Pripp, Linda Reme Sagedal

**Affiliations:** ^1^ Department of Research, Sorlandet Hospital, Kristiansand, Norway; ^2^ Department of Nutrition and Public Health, Faculty of Health and Sport Sciences, University of Agder, Kristiansand, Norway; ^3^ Department of Pediatric and Adolescents Medicine, Akershus University Hospital, Akershus, Norway; ^4^ General Practice Research Unit (AFE), Department of General Medicine, Institute of Health and Society, University of Oslo, Oslo, Norway; ^5^ Department of Public Health and Nursing, Norwegian University of Science and Technology (NTNU), Trondheim, Norway; ^6^ Department of Clinical Services, St.Olavs Hospital Trondheim University Hospital, Trondheim, Norway; ^7^ Institute of Clinical Medicine, University of Oslo, Oslo, Norway; ^8^ Department of Endocrinology, Morbid Obesity and Preventive Medicine, Oslo University Hospital, Oslo, Norway; ^9^ Oslo Centre of Biostatistics and Epidemiology, Research Support Services, Oslo University Hospital, Oslo, Norway; ^10^ Department of Obstetrics and Gynaecology, Sorlandet Hospital, Kristiansand, Norway

**Keywords:** gestational diabetes, screening, pregnancy outcomes, fasting plasma glucose, OGTT 3

## Abstract

**Background/objective:**

There is no international consensus about the optimal approach to screening and diagnosis of gestational diabetes mellitus (GDM). Fasting plasma glucose (FPG) has been proposed as an alternative universal screening test to simplify the diagnosis of GDM. We investigate the ability of the FPG to predict a 2-hour glucose value below the cut-off for GDM, thereby “ruling out” the necessity of a full OGTT and assess the proportion of GDM-related complications associated with the identified FPG level.

**Materials and methods:**

This study included secondary data from four Norwegian pregnancy cohorts (2002-2013), encompassing 2960 women universally screened with late mid-pregnancy 75g OGTT measuring FPG and 2-hour glucose. For a range of FPG thresholds, we calculated sensitivity to predict elevated 2-hour glucose, number of OGTTs needed and percentage of GDM cases missed, applying modified World Health Organization (WHO) 2013 criteria (^2013^WHO) and 2017 Norwegian criteria (^2017^Norwegian). We analyzed pregnancy outcomes for women above and below our selected threshold.

**Results:**

The prevalence of GDM was 16.6% (^2013^WHO) and 10.1% (^2017^Norwegian). A FPG threshold of 4.7 mmol/L had a sensitivity of 76% (^2013^WHO) and 80% (^2017^Norwegian) for detecting elevated 2-hour glucose, with few missed GDM cases (2.0% of those ruled out and 7.5% of all GDM cases for ^2013^WHO, and 1.1% of those ruled out and 7% of all GDM cases for ^2017^Norwegian). When excluding women with FPG <4.7mmol/l and those with GDM based on FPG, only 24% (^2013^WHO) and 29% (^2017^Norwegian) would require OGTT. Women with FPG <4.7mmol/l, including missed GDM cases, had low risk of large-for-gestational-age newborns, cesarean section and operative vaginal delivery.

**Conclusion:**

A FPG threshold of 4.7mmol/l as a first step when screening for GDM could potentially eliminate the need for OGTT in 70-77% of pregnancies. Women with FPG below this threshold appear to carry low risk of GDM-associated adverse pregnancy outcomes.

## Introduction

Gestational diabetes mellitus (GDM) is one of the most common disorders of pregnancy, responsible for several adverse outcomes in both mother and child during gestation and in the longer term (1). Despite extensive research over the past decades, there is still no consensus about the optimal approach to screening strategies and diagnostic criteria for GDM, reflected by substantial variations in clinical recommendations throughout the world (2, 3).

Although different diagnostic criteria for the identification of GDM are used, the oral glucose tolerance test (OGTT) is endorsed by all diabetes and health organizations as the “gold standard” diagnostic test for GDM. The use of OGTT in a clinical setting, however, poses several challenges. The test is poorly reproducible (4), time-consuming, and not user-friendly (5, 6), leading to a significant burden on the healthcare system in terms of infrastructure and cost. While the International Federation of Gynecology and Obstetrics strongly recommends universal testing (7) several European countries, including Norway, practice risk-factor based selective screening with the intention to identify the most severe cases of GDM and, concurrently, limit the number of OGTT’s. However, this selection process is also demanding for healthcare providers, requiring screening of about 70% of the pregnant population (8).

Fasting plasma glucose (FPG) has been proposed as an alternative universal screening test for GDM (9), as it is easy to administer, less time-consuming for patients and healthcare providers, and inexpensive (10). During the Covid-19 pandemic, in order to minimize transmission of the virus and reduce use of medical resources, several health authorities and professional bodies suggested limiting the OGTT to women with FPG above a certain threshold value, “ruling out” those with lower FPG values where the GDM risk was considered low (11). If still recommended today, these new strategies should, however, be balanced by the need to ensure the best possible pregnancy outcomes for women and their infants. To date, a number of studies have proposed FPG cut-offs to accurately rule in and rule out GDM, with wide variation amongst different geographical regions in the world (12–16), but few have evaluated pregnancy complications potentially detected or missed (13, 17).

In light of this context, we aimed to explore the use of FPG to identify women at low risk for GDM and GDM-related adverse outcomes, limiting the need for an oral glucose tolerance test (OGTT). Our primary aim was to investigate the ability of the FPG to predict a 2-hour glucose value below the cut-off for GDM, thereby “ruling out” the necessity of a full OGTT, based on two different diagnostic GDM criteria in a Norwegian pregnant population. The secondary aim was to assess the proportion of GDM-associated complications for the identified FPG level in order to evaluate whether pregnancies ruled out can be safely excluded from further post-load glucose testing.

## Material and methods

### Study population and setting

We used secondary data from four Norwegian population-based birth cohort studies with a special focus on GDM. The criteria for the selection of studies have been previously described in detail (8). Participant characteristics for all studies are summarized in **Table S1**. The merged dataset consisted of two cohort studies (18, 19) and two randomized controlled trials (RCT) (20, 21) collecting data between 2002 and 2013. The interventions in the two trials consisted of either an exercise program (20) or a combination of a physical activity component and dietary counselling (21), but these interventions demonstrated no effect on the incidence of GDM or the outcomes of LGA and caesarean section. We excluded women with multiple pregnancies, those lacking glucose values, and infants with missing birthweight. We also excluded fetal deaths, as all except one had missing OGTT and/or outcome data (Flow chart, **Figure S1**). The Norwegian Regional Ethics committees (REC) had approved each study, and the current study was approved by the REC South East. All participants provided written informed consent.

### Data collection

All women were offered a 75 g OGTT measuring fasting and 2-hour (2-h) glucose levels. In the STORK Groruddalen study (22) venous blood samples were collected in tubes containing ethylenediaminetetraacetic acid according to standardized protocols, and glucose was analyzed on site in fresh, whole EDTA blood, using HemoCue 201+ glucose analyser (Angelholm, Sweden) calibrated for plasma. In two studies (19, 23), glucose levels were measured in serum by the routine methods used at the participating hospital laboratories, and blood samples were stored at -80°C. The Fit for Delivery study measured glucose in plasma using a Cobas 6000 c501 chemistry analyzer (Roche Diagnostics) (24). Inter-essay coefficients for each study are reported in **Table S1** (CV 2.0-3.6%), and further details about the laboratory measurements can be found in the original studies.

During data collection, the diagnosis of GDM was made according to the 1999 World Health Organization (WHO) criteria (^1999^WHO) (FPG ≥7.0 mmol/l or 2-h glucose ≥7.8 mmol/l). We retrospectively applied modified 2013 WHO (^2013^WHO) diagnostic cut-offs (FPG ≥5.1 mmol/l or 2-h glucose ≥8.5 mmol/L, as 1-hour glucose was not measured in the respective studies) and the 2017 Norwegian (^2017^Norwegian) cut-offs (FPG ≥ 5.3 mmol/l or 2-h glucose ≥ 9.0 mmol/L) to the same OGTT. Women with GDM by 1999-WHO criteria were informed and referred to their general practitioner or specialist care according to protocol. Women received standard GDM treatment in accordance with either global guidelines in place at the time (25) or local guidelines specific to each hospital (treatment targets provided in **Table S1**). However, we lack specific information about the treatment provided to each woman, including whether the clinicians adhered to the guidelines. Only 12 women have been documented as receiving pharmacological treatment.

All participants provided questionnaire data, self-reported (19–21) or through interviews (18), with information on maternal age, parity, smoking status and their highest level of education. Ethnic origin was defined by the pregnant woman’s mother’s country of birth and further merged into three groups in the current study: European (predominantly Scandinavian), Middle Eastern/African, and Asian (primarily South and East Asian ethnicity). Height was measured directly on site while weight prior to pregnancy was self-reported. Pre-pregnancy body mass index (BMI) was defined as weight (kg) divided by height (m)^2^ and categorized as normal weight (≤24.9 kg/m^2^), overweight (25-29.9 kg/m^2^) and obesity (≥30 kg/m^2^).

### Pregnancy and delivery outcome data

Outcome data collected at the time of birth were birthweight (grams), gestational age at birth, delivery method (normal vaginal delivery, cesarean section (planned or emergency), operative vaginal delivery (vacuum extraction or forceps)), preeclampsia or severe hypertensive disorder, and preterm delivery (<37 weeks). As in clinical practice in Norway, LGA (sex and gestational age-specific birthweight >90th percentile) was calculated using Norwegian national references (26), while macrosomia for the present study was defined as birthweight >4000 g.

### Statistical analyses

The area (AUC) under the receiver operating characteristic (ROC) curve was used to analyze the discriminative power of FPG to predict an elevated 2-h glucose value, using the modified ^2013^WHO criteria and the ^2017^Norwegian criteria. Elevated 2-h glucose was used instead of the diagnosis of GDM since the latter also includes those diagnosed based on FPG. Using standard definitions, we assessed diagnostic accuracy measures such as sensitivity, specificity, and negative predictive value (NPV) of a range of threshold values of FPG (varying from 4.4 to 5.0 mmol/l). The number of OGTTs needed was analyzed after excluding women who had GDM based on FPG alone (FPG≥5.3 mmol/l or ≥5.1 mmol/l, depending on the diagnostic criteria). In addition, we calculated the proportion of missed GDM cases (women with GDM according to the 2-hour glucose but “ruled out” and excluded from the OGTT because of the specified FPG threshold). In the process of selecting the “optimal” FPG threshold, options that demonstrated good/acceptable sensitivity were considered (27). The thresholds were then reviewed individually according to diagnostic needs and clinical usefulness, with particular emphasis on the number of required OGTTs and missed GDM cases.

Characteristics of the women were categorized by FPG-status, and the groups were compared using X^2^ statistic for categorical data and ANOVA for continuous variables. Data are presented as frequencies and percentages for categorical variables and mean and standard deviations (SD) for continuous variables.

To examine the risk of pregnancy complications among missed GDM cases (GDM according to the 2-hour glucose, but potentially excluded from the OGTT based on low FPG), we stratified women further into two groups: FPG below or FPG at/above the proposed threshold (4.7 mmol/l). For both strata, multivariable logistic regression models were performed for the pregnancy outcomes LGA, cesarean section and operative vaginal delivery, with elevated 2-h glucose (categorized as less than or at/above 9.0 mmol/l) as the main exposure. We adjusted for pre-specified confounders such as maternal age, pre-pregnancy BMI, ethnicity, parity, cohort, smoking and gestational age at birth. The effect estimates for 2-h glucose (less than or at/above 9.0 mmol/l) is presented as odds ratios (OR) with 95% confidence intervals (CI). The level of significance was set as 0.05. Statistical analyses were performed using statistical package IBM SPSS (version 23.0. Armonk, NY: IBM Corp).

## Results

Of the 2970 women included in the present study, 16.6% fulfilled the modified ^2013^WHO criteria for GDM, while 10.1% met the ^2017^Norwegian criteria. More than 80% of all GDM cases were identified by elevated FPG, both by the modified ^2013^WHO (≥5.1 mmol/L) and the ^2017^Norwegian (≥5.3 mmol/L) criteria, while 16.0% and 17.6% were identified by elevated 2-h glucose alone [^2013^WHO (≥8.5 mmol/L) and ^2017^Norwegian criteria (≥9.0 mmol/L)] (**Table S2**).

### The ability of FPG to ‘rule-out’ the need for a full OGTT

**Figure 1:**
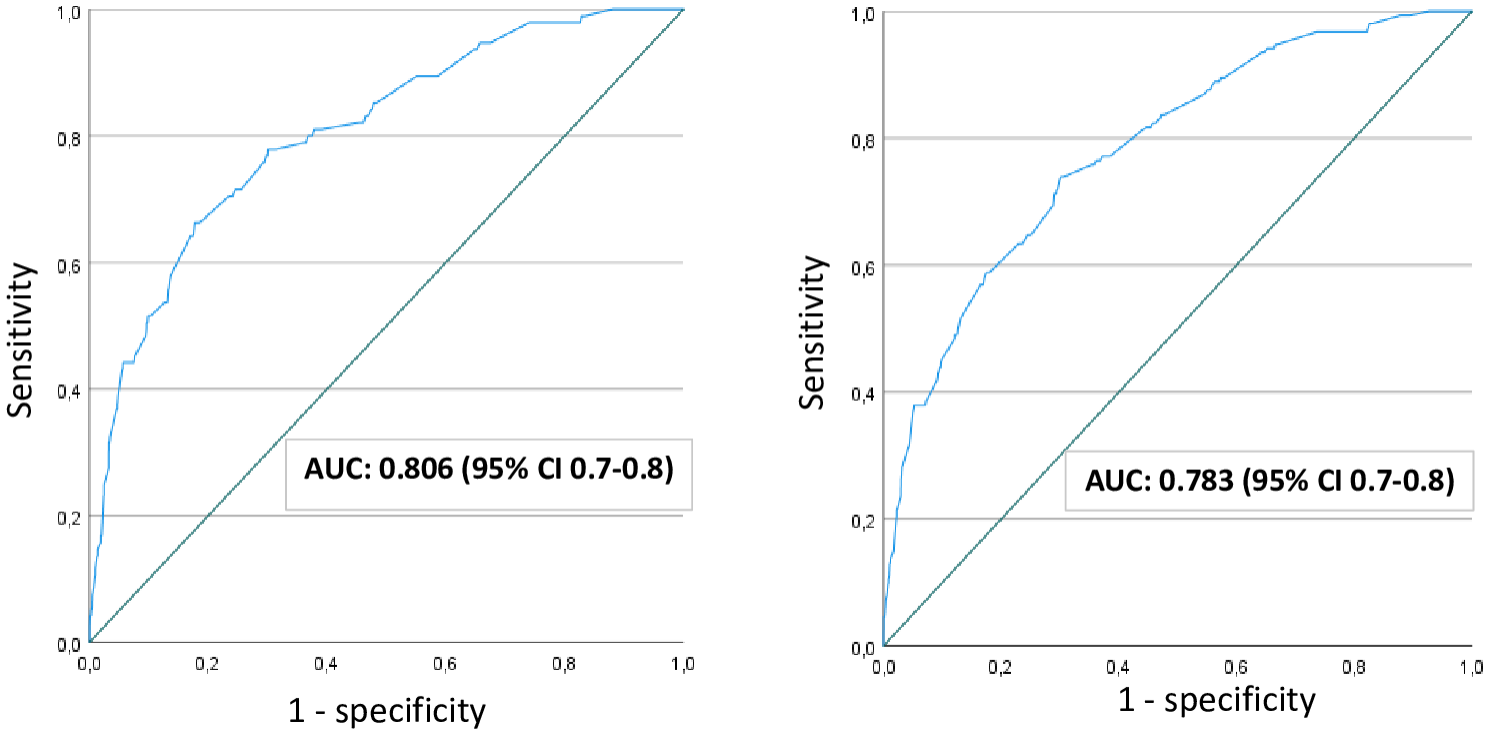
ROC curve to assess the performance of fasting plasma glucose to predict elevated 2-hour glucose, applying 2017-Norwegian criteria (left) and applying modified 2013-WHO criteria (right). AUC, area under the curve; CI, confidence interval; WHO, World Health Organization.

The ROC curves along with the AUC quantifying the performance of the FPG to predict an elevated 2-h glucose (diagnostic for GDM) were assessed graphically (**Figure 1**). The AUC was 0.81 (95% CI 0.76-0.85) using the ^2017^Norwegian criteria and slightly lower (0.78, 95% CI 0.75-0.82) when the modified ^2013^WHO criteria were used to define GDM. A separate ROC analysis for women with non-European background gave an AUC of 0.70 (95% CI 0.6-0.8) using the ^2017^Norwegian criteria (**Figure S2**).

**Table 1A** lists a range of threshold values for FPG with the associated sensitivity, specificity, and negative predictive value (NPV) using the Norwegian criteria. As the cut-off value rises, the sensitivity of the screening test decreases and the specificity increases. Conversely, a lower FPG threshold has high sensitivity and identifies most women with GDM but an excessive number of women without GDM will need to undergo the OGTT due to the corresponding poor specificity. Based on test properties (27) and careful clinical judgment the threshold value 4.7 mmol/L for FPG was selected, as this threshold had an acceptable sensitivity (78.9%) to predict elevated 2-hour glucose and appeared to offer the best trade-off to limit the number of missed GDM cases while avoiding unnecessary OGTTs. In total, 1855 women (62.5%) had FPG below this threshold and could potentially be ‘ruled-out’ as non-GDM. Of these women, 20 (1.1% of those ruled out and 6.6% of all GDM cases) had an elevated 2-hour glucose value, and would hence be “misclassified” as non-GDM, with the NPV being 98.9%. Of the remaining 1111 women with FPG above the 4.7mmol/l threshold, 248 (8.4% of the entire cohort) had FPG ≥5.3 mmol/l, i.e. GDM according to the ^2017^Norwegian diagnostic criteria (**Table 2**). Thus, only the remaining 864 (29.1% of the entire cohort) would have to undergo the complete OGTT.

**Table 1A:** Overview of the sensitivity of different thresholds of FPG to the need for an OGTT to screen for GDM (Norwegian 2017 criteria).

Threshold FPG (mmol/l)	No. of women below threshold, n (%)	No. of OGTT needed*, n (%)	No. of GDM cases missed^, n (%)°	Sensitivity for 2-h glucose, % n/N	Sensitivity for GDM, % n/N	Specificity, %	NPV (%)
**4,4**	1051 (34,2)	1703 (57.4)	6 (1.9)	93,7 (89/95)	98,0 (295/301)	35.2	99,4
**4,5**	1298 (43,8)	1420 (47.9)	10 (3.3)	89.5 (85/95)	96,7 (291/301)	44.9	99.2
**4,6**	1586 (53,5)	1132 (38.1)	17 (5.6)	82.1 (78/95)	94,4 (284/301)	54.7	98.9
**4,7**	1855 (62.5)	863 (29.0)	20 (6.6)	78,9 (75/95)	93,3 (281/301)	63.9	98,9
**4,8**	2052 (69,2)	666 (22.4)	23 (7.6)	75,8 (72/95)	92,3 (278/301)	70.7	98,9
**4,9**	2222 (74,9)	526 (17.7)	28 (9.3)	70.5 (67/95)	90,0 (273/301)	76.7	98,7
**5.0**	2414 (81,4)	304 (10.2)	34 (11.3)	64.2 (61/95)	88,7 (267/301)	82.9	98,6

*Excluding women diagnosed with FPG ≥5.3 mmol/L (248 women).

^Women with FPG below stated threshold but 2-hour glucose above diagnostic criteria (>9.0 mmol/l), i.e. false negative cases.

FPG, fasting plasma glucose; OGTT, oral glucose tolerance test; GDM, gestational diabetes mellitus; NPV, negative predictive value; no, number.

n: total number of cases; %: percentage of the total study cohort; °: percentage of total GDM cases.

**Table 1B:** Overview of the sensitivity of different thresholds of FPG to the need for an OGTT to screen for GDM 2013 WHO criteria.

Threshold FPG (mmol/l)	No. of women below threshold, n (%)	No. of OGTT needed*, n (%)	No. of GDM cases missed^, n (%)°	Sensitivity for 2-h glucose, % n/N	Sensitivity for GDM, % n/N	Specificity, %	NPV (%)
**4,4**	1051 (34,2)	1536 (51.7)	10 (2.0)	93.5 (143/153)	98,0 (484/494)	35.8	99.0
**4,5**	1298 (43,8)	1254 (42.2)	20 (4.0)	86.8 (133/153)	95,9 (474/494)	45.4	98.5
**4,6**	1586 (53,5)	965 (32.5)	28 (5.7)	81.7 (125/153)	94,3 (466/494)	55.4	98.2
**4,7**	1855 (62.5)	697 (23.4)	37 (7.5)	75.8 (116/153)	92,5 (457/494)	64.6	98.0
**4,8**	2052 (69,2)	499 (16.8)	47 (9.5)	69.3 (106/153)	90,5 (447/494)	71.3	97.7
**4,9**	2222 (74,9)	329 (11.0)	56 (11.3)	63.4 (97/153)	88,7 (438/494)	77.0	97.5
**5.0**	2414 (81,4)	137 (4.6)	66 (13.4)	56.9 (87/153)	86,6 (428/494)	83.5	97.3

*Excluding women diagnosed with FPG ≥5.1 mmol/L (415 women).

^Women with FPG below listed threshold but 2-hour glucose above diagnostic criteria (>8.5 mmol/l), i.e. false negative cases.

FPG, fasting plasma glucose; OGTT, oral glucose tolerance test; GDM, gestational diabetes mellitus; NPV, negative predictive value; no, number.

n: total number of cases; %: percentage of the total study cohort; °: percentage of total GDM cases.

**Table 2:** Comparison of characteristics and pregnancy outcomes between women with fasting plasma glucose below and at/above 4.7 and ≥5.3/5.1 mmol/l (2017 Norwegian cut offs and 2013 WHO cut offs).

Characteristics	Total	<4.7 mmol/L	Norwegian 2017 Criteria			2013 WHO criteria		
			4.7-5.2 mmol/L	≥5.3 mmol/L	*p*	4.7-5.0 mmol/L	≥5.1 mmol/L	*p*
n	2967	1855 (62.5)	864 (29.1)	248 (8.4)		697 (23.5)	415 (14.0)	
Maternal age (years)	30.0 (4.4)	29.9 (4.2)	30.5 (4.5)	30.7 (5.0)	0.001	30.4 (4.4)	30.6 (5.0)	0.001
Pre-pregnancy BMI (kg/m²)	23.7± 3.9	22.9 (3.3)	24.5 (4.1)	26.5 (5.7)	0.000	24.3 (4.0)	25.9 (5.3)	0.000
normalweight	2127 (71.6)	1457 (78.5)	552 (63.9)	116 (46.8)		455 (65.3)	213 (51.3)	
overweight	610 (20.5)	315 (17.0)	225 (26.0)	69 (27.8)		175 (25.1)	119 (28.7)	
obesity	233 (7.8)	83 (4.5)	87 (10.1)	63 (25.4)		67 (9.6)	83 (20.0)	
Ethnicity					0.000			0.000
European	2570 (86.6)	1705 (91.9)	708 (81.9)	157 (63.3)		584 (83.8)	281 (67.7)	
Middle East/African	174 (5.9)	68 (3.7)	68 (7.9)	38 (15.3)		48 (6.9)	58 (14.0)	
Asian	223 (7.5)	82 (4.4)	88 (10.2)	53 (21.4)		65 (9.3)	76 (18.3)	
Primipara, n (%)	1814 (61.1)	1174 (63.3)	517 (59.8)	123 (49.6)	0.000	424 (60.8)	216 (52.0)	0.000
Education, n (%)					0.000			0.000
Primary or less	145 (4.9)	61 (3.3)	51 (5.9)	34 (13.7)		33 (4.7)	52 (12.5)	
High school education	637 (21.4)	329 (17.7)	231 (26.7)	80 (32.3)		184 (26.4)	127 (30.6)	
Higher education	2180 (73.4)	1465 (79.0)	582 (67.4)	134 (54.0)		480 (68.9)	236 (56.9)	
Current smoker, n (%)	80 (2.8)	41 (2.3)	30 (3.7)	9 (4.0)	0.064	24 (3.7)	15 (3.9)	0.063
Fasting glucose at OGTT (mmol/L)	4.6± 0.5	4.3 (0.2)	4.9 (0.1)	5.7 (0.4)	0.000	4.8 (0.4)	5.4 (0.4)	0.000
2-hour glucose at OGTT (mmol/L)	6.1± 1.3	5.7 (1.1)	6.4 (1.3)	7.2 (1.6)	0.000	6.3 (1.2)	7.0 (1.5)	0.000
Gestational age at OGTT (weeks)	30.8 ± 2.5	31.4 (2.6)	30.1 (2.1)	29.5 (2.0)	0.000	30.2 (2.1)	29.6 (2.0)	0.000
GDM treatment								
Insulin/metformin	12 (0.4)	0	4	8		4	8	
*Delivery*								
Gestational age at delivery (weeks)	39.8 (1.6)	38.9 (1.5)	39.7 (1.6)	39.5 (1.6)	0.009	39.7 (1.6)	39.6 (1.6)	0.023
Birthweight, gram	3520 (522)	3485.7 (501)	3567.9 (529)	3607.1 (560)	0.000	3556.8 (532)	3610.1 (542)	0.000
LGA, n (%)	230 (7.7)	117 (6.3)	73 (8.4)	40 (16.1)	0.000	60 (8.6)	53 (12.8)	0.000
Macrosomia >4000g, n (%)	507 (17.1)	274 (14.8)	168 (19.4)	65 (26.2)	0.000	133 (19.1)	100 (24.1)	0.000
Total cesarean section, n (%)	446 (15.0)	244 (13.2)	143 (16.6)	60 (24.2)	0.000	113 (16.2)	90 (21.7)	0.000
Emergency cesarean section, n (%)	298 (10.1)	171 (9.2)	85 (9.8)	42 (16.9)	0.000	68 (9.8)	59 (14.2)	0.000
Preterm birth	108 (3.6)	67 (3.6)	28 (3.2)	13 (5.2)	0.331	22 (3.2)	19 (4.6)	0.470
Preeclampsia	98 (3.6)	62 (3.7)	27 (3.3)	9 (3.8)	0.861	22 (3.4)	14 (3.6)	0.908
Operative vaginal delivery, n (%)	386 (13.0)	242 (13.1)	109 (12.6)	34 (13.7)	0.893	91 (13.1)	52 (12.5)	0.958

P values refer to comparison between the three groups using ANOVA.

FPG, fasting plasma glucose; OGTT, oral glucose tolerance test; BMI, body mass index; GDM, gestational diabetes; WHO, World health organization; LGA, large for gestational age.

Similar results were found for the modified ^2013^WHO criteria (**Table 1B**), although sensitivity to predict elevated 2-hour glucose was slightly reduced (75.8%), and the number of missed GDMs cases was slightly higher (2.0% of those ruled out and 7.5% of all GDM cases) for the same FPG threshold. The proportion of women requiring further evaluation to define their GDM status on the basis of FPG 4.7-5.0 mmol/L (number of OGTT needed) was, on the other hand, slightly lower using these criteria (23.5%).

Thus, if the FPG was offered to all women and a FPG threshold of 4.7 mmol/l was used to decide whether the OGTT was needed or not, 70.9% of women in the cohort would not require further testing when using the ^2017^Norwegian diagnostic thresholds, and 76.5% when using the modified ^2013^WHO criteria.

### Comparison of women below or above the selected FPG threshold

**Table 2** presents maternal characteristics and the proportion of pregnancy outcomes found among women classified as low FPG (<4.7 mmol/l), indeterminate FPG (4.7-5.2/5.0 mmol/l) and elevated FPG (FPG ≥5.3/5.1 mmol/l) according to the ^2017^Norwegian criteria and ^2013^WHO criteria, respectively. Women in the low FPG group had the lowest pre-pregnancy BMI. They also had the highest proportion of primiparas and the lowest proportion of women with a non-European ethnicity. Furthermore, the lowest proportion of LGA, macrosomia (>4000g) and total cesarean section was observed in women with FPG <4.7 mmol/L and the highest proportion in the elevated FPG groups. None of the women with FPG <4.7 mmol/l and GDM by ^1999^WHO criteria received insulin or other antidiabetic medication.

**Table 3** reports the proportion of pregnancy complications amongst women with GDM based on an elevated 2-hour glucose value (≥9.0 mmol/L), after dividing the sample into those below or at/above the FPG threshold of 4.7 mmol/l. For women with FPG ≥4.7 mmol/l [including those meeting current GDM criteria (FPG ≥5.3 mmol/L)], 2-h glucose ≥9.0 mmol/l was associated with higher risk for LGA (OR 2.61; 95%CI 1.37-4.95) but not for cesarean section and operative vaginal delivery. For women with FPG <4.7 mmol/l, who would not be offered an OGTT according to the proposed strategy, 2-h glucose ≥9.0 mmol/l was not associated with an increased risk for any of these outcomes.

**Table 3:** Pregnancy outcomes stratified according to fasting plasma glucose (2017 Norwegian criteria).

Delivery outcomes	Total	<4.7 mmol/L			≥4.7 mmol/L		
2-hour glucose values		<9.0	>9.0	aOR* (95% CI)	<9.0	>9.0	aOR* (95% CI)
n	2967	1835	20		831	33	
Birthweight, gram	3520 (522)	3488	3243		3564	3663	
LGA, n (%)	230 (7.7)	116 (6.3)	1 (5.0)	1.01 (0.12-7.91)	67 (8.1)	6 (18.2)	**2.612 (1.37-4.95)**
Macrosomia >4000g, n (%)	507 (17.1)	237 (14.9)	1 (5.0)		161 (19.4)	7 (21.2)	
Total cesarean section, n (%)	446 (15.0)	240 (13.1)	4 (20.0)	1.20 (0.38-3.84)	137 (16.5)	6 (18.2)	1.040 (0.58-1.85)
Preterm birth	108 (3.6)	65 (3.5)	2 (10.0)		27 (3.2)	1 (3.0)	
Preeclampsia	89 (3.6)	61 (3.7)	1 (5.6)		27 (3.5)	0	
Operative vaginal delivery, n (%)	386 (13.0)	167 (9.1)	2 (10.0)	1.018 (0.37-2.75)	84 (10.1)	3 (9.1)	1.087 (0.65-1.80)

Significant values in bold.

*Adjusted for age, prepreg BMI, parity, ethnicity, cohort, smoking (LGA only) and gestational age at birth (ceserean section and operative delivery).

## Discussion

### Main findings

Using data from a Norwegian sample offered universal mid-pregnancy GDM screening, we found that a FPG threshold of 4.7 mmol/L demonstrated an acceptable sensitivity of 76-80% to predict an elevated 2-hour glucose value (using modified ^2013^WHO and ^2017^Norwegian criteria respectively); 63% of participants had FPG below this threshold and could be “ruled-out” from further testing regardless of criteria used. Importantly, this group appears to carry a low risk of a range of pregnancy complications commonly associated with GDM. Furthermore, because FPG is included in the diagnostic criteria, we could identify (“rule-in”) over 80% of GDM cases using FPG alone in our sample. This implies that if a rule-in/rule-out approach was used, OGTT would be needed in only 24-29% of our population, i.e. only those with FPG in the range 4.7-5.0/5.2 mmol/L.

### Interpretation

FPG thresholds previously suggested as the preferred cut-off to avoid unnecessary OGTT’s include 5.0 mmol/l in Mexican adolescents (14), 4.8 mmol/l in Swedish women (16), 4.4 mmol/l in both an Arab (12) and Chinese population (15), and 4.3 mmol/l in studies from South Asia (28) and Belgium (13). The diagnostic performance of FPG as a screening test is dependent on the population tested, GDM prevalence and GDM criteria used (29, 30). In addition, the determination of ideal test sensitivity and specificity requires judicious assessment of harms related to missed diagnosis as well as burdens associated with large-scale testing. A low FPG threshold will have high sensitivity and identify most women with GDM but an excessive number of women without GDM will need to undergo the OGTT due to corresponding poor specificity, putting pressure on health services and medicalizing low-risk pregnancies. Similar to our findings, a recent Australian study concluded that FPG ≥4.7 mmol/l had the best sensitivity and specificity for abnormal OGTT results (31), and this preliminary test was employed in Australia during the Covid-19 pandemic.

Although more than 70% of OGTT’s could be avoided by the proposed strategy in our study, applying either ^2013^WHO or ^2017^Norwegian criteria, 7-8% of GDMs identified with universal OGTT would be missed. Others have reported higher rates of “missed GDM” for the same threshold. Van Gemert et al. compared the use of a preliminary FPG ≥4.7 mmol/l to universal OGTT, reporting that 29% of women who would otherwise be diagnosed with GDM by ^2013^WHO criteria could be missed (11). The contrasting finding in this study may at least to some degree be explained by additional measurements of 1-hour glucose values which we lacked. Nevertheless, recognizing and diagnosing GDM is essential, as management of GDM has been associated with reduced maternal, fetal and newborn complications (32–34). Furthermore, the identification of GDM provides a valuable opportunity to assess the women’s future risk of diabetes and implement preventive measures, a possibility that would remain beyond reach without proper identification.

In our cohort, 82-84% of all GDM cases were identified based on FPG, making the idea of entirely abandoning an assessment of post-load glycemia rather appealing. A single FPG test offered to all pregnant women is a simple and low-cost option to diagnose GDM. However, the proportion of women diagnosed by FPG in our study was much higher than reported by others, including the multinational HAPO study, where 55% were diagnosed with GDM by FPG, using ^2013^WHO criteria (35). Given the wide variability in the percentage of women diagnosed exclusively by FPG, probably explained by factors such as ethnicity and varying rates of obesity (35, 36) continued use of OGTT seems indicated.

Few previous studies have addressed whether pregnancies with FPG below a proposed threshold are in fact associated with low rates of GDM-associated complications. McIntyre et al. examined the outcomes associated with the Australian Covid-19 model of limiting GDM testing to those with FPG ≥4.7 mmol/l, using a subset of the Hyperglycemia and Adverse Pregnancy Outcome (HAPO) study (37). Broadly consistent with our findings, participants with FPG <4.7 mmol/L had lower rates of pregnancy complications than those above this threshold. A recent Belgian study assessed pregnancy outcomes for a FPG threshold of 4.3 mmol/l, finding a better metabolic profile and low incidence of adverse outcomes below this cut-off (13), but the clinical relevance may be limited as this threshold excludes few women from testing. Our findings indicate that women with FPG <4.7 mmol/l had a better metabolic profile, with less overweight/obesity, compared to women with higher FPG. In addition, pregnancies with FPG <4.7 mmol/l had low rates of LGA, macrosomia and total cesarean section, indicating that these women can safely continue routine care. However, long-term health risks in these women and their children, particularly related to type 2 diabetes, are unknown.

We have previously reported that selection criteria for BMI and age currently used in Norway would result in recommending OGTT to about 70% of women with European ethnicity in our sample (8). The results from the current study lend support to the universal use of FPG as an alternative to risk-profiling for selectively offering the OGTT, with the potential to limit the use of OGTT to less than 30% of all pregnancies and achieve similar sensitivity of about 80%. Additionally, it may avoid potentially stigmatizing selection based on age, BMI and ethnicity.

However, the proposed screening strategy requires certain logistics to be in place in order to make the implementation successful. Ideally, the fasting venous sample would have to be analyzed without delay by a measure with acceptable validity and reliability and at the same facility. This should be followed by an immediate decision as to whether a full OGTT is required, thereby avoiding prolonged waiting time and enabling women to complete the test on the same day as the fasting blood test. Moreover, our study is centered on GDM diagnosis made late in pregnancy. In light of a recent RCT indicating potential benefits of early screening (38), the matter of early versus late screening also warrants further consideration and exploration.

### Strengths and weaknesses

Our study has several strengths. We merged previously collected maternal and offspring data from four cohorts, allowing more powerful and flexible analyses. Moreover, there is no pre-selection bias as an OGTT was offered to all included women. Importantly, most previous studies that have explored the performance of FPG provide limited or no information on adverse pregnancy outcomes.

The main limitation of our study is that glucose results were not blinded and women with GDM diagnosed by ^1999^WHO criteria were routinely treated. This implies that conclusions drawn about likely clinical outcomes for women classified as “missed GDMs” may be inaccurate, as patients had a known diagnosis and received care, although none of these women received pharmacological treatment. Nonetheless, our results are comparable to those of McIntyre et al., which used a population blinded to OGTT results in their retrospective analysis of FPG and pregnancy outcomes (17). Additionally, our sample had slightly lower rates of obesity than our background population (7.8% vs. 12-12.5% nationally in 2007-2013) (39). This may affect the prevalence of GDM and its associated outcomes, and the proportion of GDM identified by FPG. The pre-analytical processing and measurement of glucose is critical for accuracy in GDM diagnosis. Differences in sampling and analytical procedures across studies (one study used point-of-care whole blood glucose and two studies used serum) is another weakness of the current study, potentially affecting the uniformity of GDM diagnosis. Despite high precision for glucose measurement in all studies (small CV’s), we cannot rule out that minor bias may have been introduced. Furthermore, the 1-hour glucose was not measured in any of the four cohorts. In the HAPO study population, 5.7% additional GDMs were identified by the 1-hour values when using the ^2013^WHO criteria (40), and a higher prevalence of GDM in our study could be expected if such data were available. Finally, very few women were diagnosed based on 2-hour glucose alone (16-18% of GDM cases) which makes analysis of women in this category difficult due to power limitations. Importantly, the proposed approach may not circumvent as many OGTTs in other populations as indicated by our study and such differences may need to be considered when extrapolating our results to other settings (i.e. to more high-risk populations). If implemented, this screening procedure should be followed by careful assessment of any potential increase in unwanted pregnancy outcomes. Further studies are needed to compare current risk-factor based screening strategies with a “rule-in, rule-out” procedure with focus on birth outcomes and cost-effectiveness.

## Conclusion

Our study suggests that a two-step approach to GDM screening, with an initial universal FPG and exclusion of low-risk women from further testing, could potentially limit the use of OGTT to less than 30% of all pregnancies. A FPG threshold of 4.7 mmol/l appears to identify women at low risk of both elevated 2-hour glucose and GDM-associated adverse pregnancy outcomes. Additional studies are needed to validate our findings and confirm the safety of this screening approach, including long-term health outcomes, especially in populations where a higher proportion of women are diagnosed with GDM from post-load values.

## Data availability statement

The data analyzed in this study is subject to the following licenses/restrictions: The datasets analyzed during the current study are not publicly available due to the dataset containing clinical data which cannot be shared publicly, and as the study is part of a PhD work. The data are available from the corresponding author on reasonable request. Requests to access these datasets should be directed to Line Sletner, line.sletner@medisin.uio.no.

## Ethics statement

The studies involving humans were approved by Norwegian Regional Ethics Committee South East. The studies were conducted in accordance with the local legislation and institutional requirements. The participants provided their written informed consent to participate in this study.

## Author contributions

AR: Data curation, Formal Analysis, Investigation, Methodology, Writing – original draft, Writing – review & editing. LS: Supervision, Data curation, Writing – review & editing. AJ: Conceptualization, Investigation, Data curation, Supervision, Writing – review & editing. NØ: Supervision, Data curation, Writing – review & editing. SS: Data curation, Writing – review & editing. EQ: Data curation, Writing – review & editing. AP: Formal analysis. LRS: Supervision, Data curation, Writing – review & editing.

## Supplementary material

Supplementary Figure 1 Flowchart of included studies and excluded participants from each study.

Supplementary Figure 2 ROC curve to assess the performance of fasting plasma glucose to predict elevated 2-hour glucose in women with non-European background **(A)** applying ^2017^Norwegian criteria **(B)** applying modified ^2013^WHO criteria AUC, area under the curve; CI, confidence interval; WHO, World Health Organization.
