# Supplementary figures and images for: Employing fasting plasma glucose to safely limit the use of oral glucose tolerance tests in pregnancy: a pooled analysis of four Norwegian studies
Source: Front Endocrinol (Lausanne). 2023 Nov 30;14:1278523. doi: 10.3389/fendo.2023.1278523 (PMC10720624; doi:10.3389/fendo.2023.1278523)

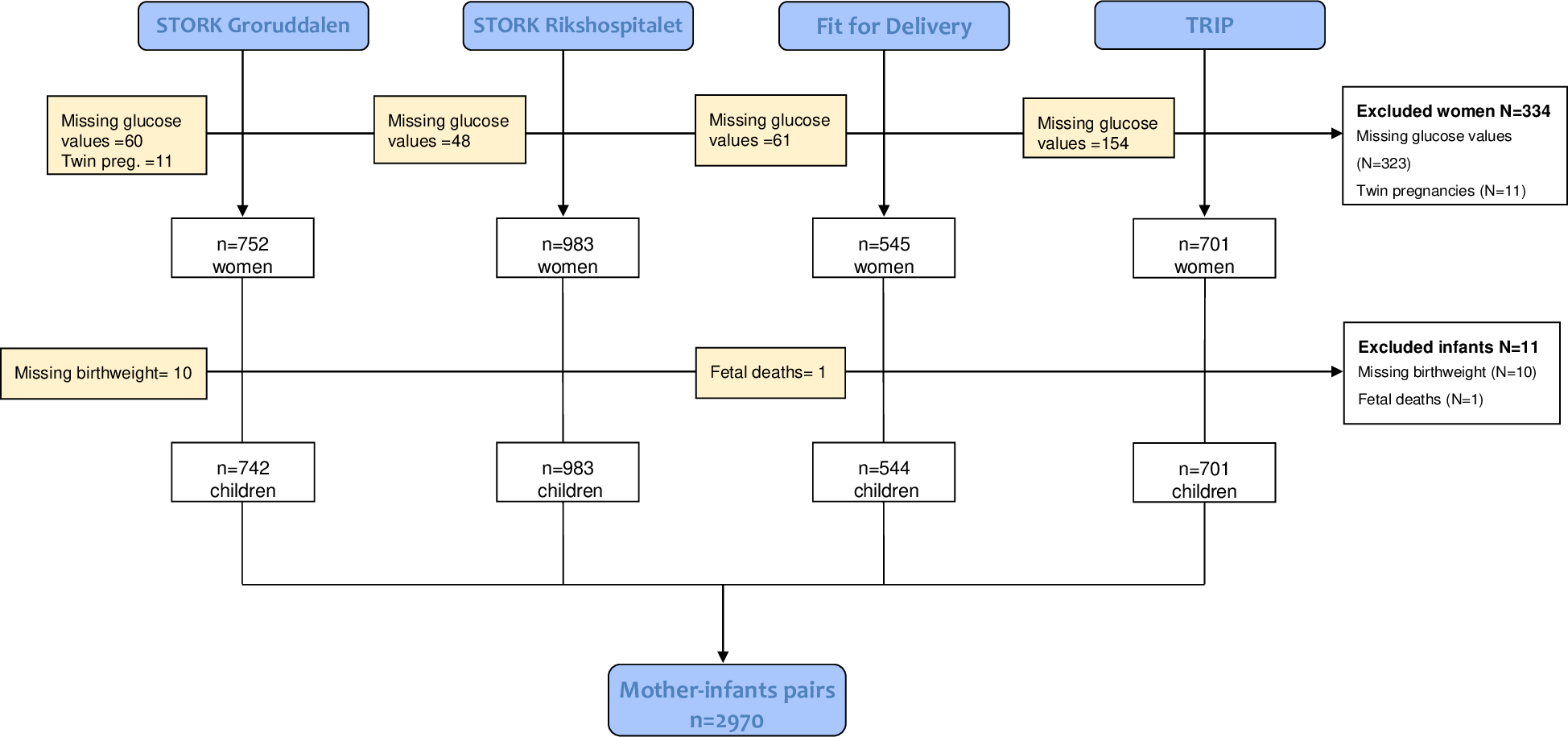

Supplement: Supplementary Figure 1 — Flowchart of included studies and excluded participants from each study. [file Image_1.tif]

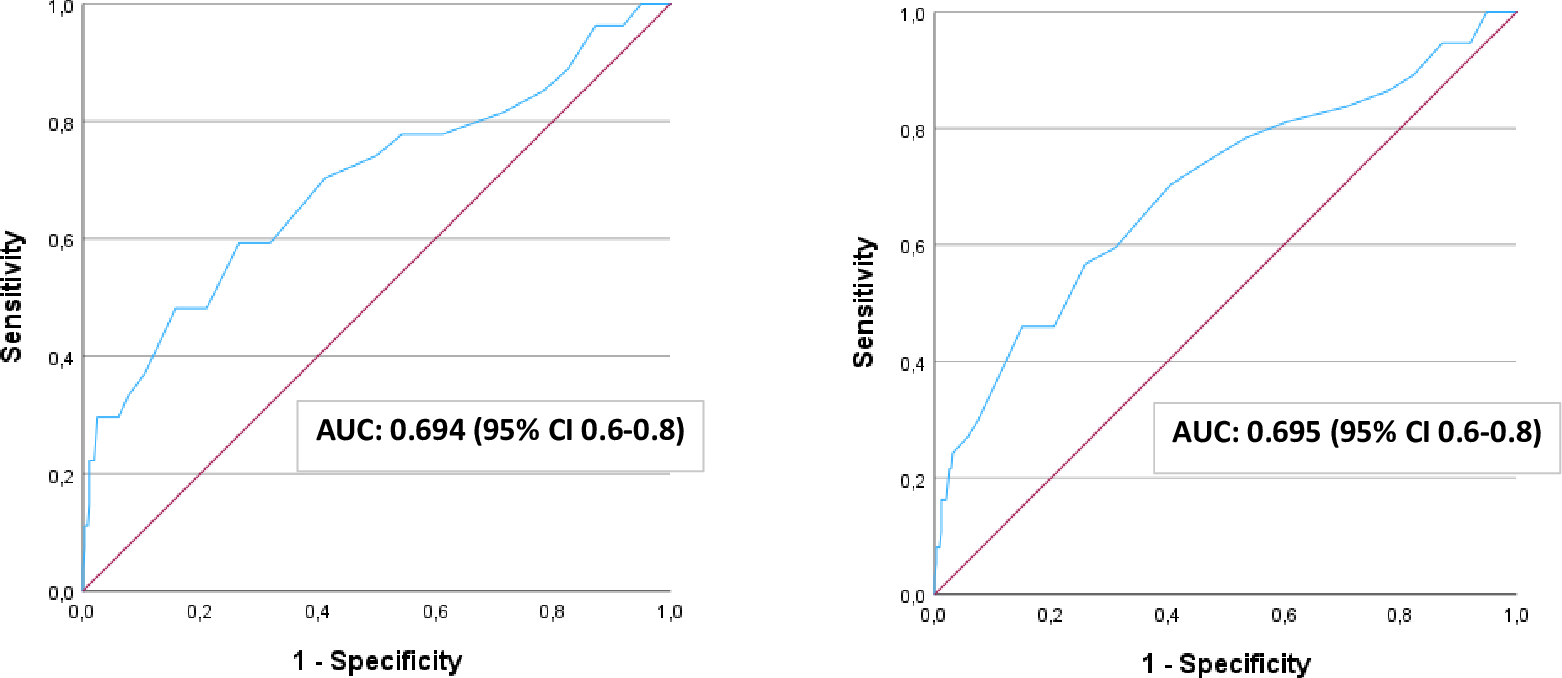

Supplement: Supplementary Figure 2 — ROC curve to assess the performance of fasting plasma glucose to predict elevated 2-hour glucose in women with non-European background (A) applying 2017Norwegian criteria (B) applying modified 2013WHO criteria AUC, area under the curve; CI, confidence interval; WHO, World Health Organization. [file Image_2.tif]
